# Manufacture of Sustainable Clay Bricks Using Waste from Secondary Aluminum Recycling as Raw Material

**DOI:** 10.3390/ma11122439

**Published:** 2018-12-02

**Authors:** Eduardo Bonet-Martínez, Luis Pérez-Villarejo, Dolores Eliche-Quesada, Eulogio Castro

**Affiliations:** 1 Department of Chemical, Environmental and Materials Engineering, Universidad de Jaén, Campus Las Lagunillas, s/n, 23071 Jaén, Spain; eduardobonet@gmail.com (E.B.-M.); lperezvi@ujaen.es (L.P.-V.); deliche@ujaen.es (D.E.-Q.); 2 Center for Advanced Studies in energy and Environment (CEAEMA), Universidad de Jaén, Campus Las Lagunillas, s/n, 23071 Jaén, Spain

**Keywords:** aluminum filter dust, ecofriendly bricks, waste management, mechanical properties, inertization

## Abstract

The aluminum recycling industry produces aluminum filter dust (AFD), a waste byproduct of the aluminum recycling process composed mainly of aluminum oxide in a percentage between 60–70%, 8% calcium oxide, almost 15% sodium chloride, and between 5–10% potassium chloride. Due to its aluminum content, this waste can be used as a raw material in the manufacture of ceramic bricks, at the same time reducing the environmental impact produced in landfill. In this work, the partial substitution of a clay mixture (40% black, 30% red, and 30% yellow clay) by different proportions of AFD in the range 0–25 wt % for the production of fired clay brick was studied. The raw materials, clays, and waste were characterized by XRF and XRD. The brick specimens were fired at 950 °C and their physical and mechanical properties, such as water absorption, water suction, loss of ignition, linear shrinkage, bulk density, and compressive strength, were analyzed. The more relevant results were obtained with the addition of up to 20 wt % AFD, obtaining bricks with physical properties comparable to pure clay-based bricks used as a reference and better compressive strength and thermal conductivity due to the balance between the melting and pore-forming effects of the waste. These sustainable bricks also comply with the regulations of heavy metals leached to the environment, as indicated by the leaching test.

## 1. Introduction 

One of the great challenges that humanity must face today is to achieve a feasible and viable solution addressing the waste generated by industry. The principles of sustainability and circular economy, as well as respect for the environment, should be a priority in current construction. It is therefore interesting to use different industrial waste in the manufacture of different construction materials, such as ceramic clay bricks. The ceramic industry presents enormous potential for the reuse and recovery of industrial waste [1,2,3,4,5,6,7]. The Spanish structural ceramic sector is the largest European producer, with about 30 million tons in 2007. Since 2008, coinciding with the economic crisis, the ceramic sector has experienced a continuous decrease in production data, with a recovery in the last two years of 6.10% [8]. 

Aluminum is the most consumed nonferrous metal in the world, with a current annual consumption at 24 million tons, and is one of the most abundant materials in the Earth’s crust [9]. One of the advantages of aluminum is that recycling does not damage the structure of the metal and can be recycled indefinitely without detracting from its qualities [10]. Secondary aluminum is the type that originates from the fusion processes of aluminum products that have reached the end of their useful life cycle [11]. Obtaining secondary aluminum presents a series of advantages over primary aluminum, since there is a saving in energy consumption between 5% and 20% [12]. There is no extraction of minerals and, therefore, there is a saving of raw materials, natural resources [13], and the environmental impact is lower. The recycling and recovery process that takes place at the Befesa Aluminio S.L., located in Valladolid (Spain), consists of two main processes: One of initial fusion of the materials in rotary type ovens, and another of refining the final product in reverberatory type ovens. The raw materials (waste from the aluminum sector) selected and treated are melted in the rotary kilns, to which also certain amounts of salt are added as a flux and protector of the molten aluminum. The gases produced during the fusion process are evacuated through purification systems, consisting of cooling systems and bag filters, where the solid particles are retained [14]. The filter dust that is produced as a consequence of the treatment of the combustion gases passes through these purification systems. The waste is a powder of very fine granulometry (<100 μm) that has a variable chemical composition, depending on the composition and quality of the secondary raw material precursor, as well as the collection system and classification method [15]. Demand for secondary aluminum over the next few years will experience an annual growth rate of around 5%, twice the rate of growth of primary aluminum (2.4%) [16]. Therefore, the generation of waste from the second melting of aluminum will also grow, so it is necessary to work on trying to address this type of waste, because otherwise the landfills will be filled and there will be no space to deposit them [17]. The characterization and incorporation of waste from the aluminum industry as raw material in the manufacture of construction materials has been studied by different authors. Huang et al. [18] characterized the mineral phases, metal content, and metal leachability of different salt cake from secondary aluminum production collected from different facilities across the U.S. Kumar Mandal et al. [19] investigated the possible use of aluminum plant waste for the preparation of insulating bricks. The bricks were manufactured by mixing fly ash and red mud in different proportions with sawdust, studying the influence of the firing temperature. The results indicated that bricks fired at 1100 °C and incorporated 7.5% sawdust and 40% red mud in fly ash to comply with the regulation criteria for insulation bricks. Gil et al. [20] investigated the management and valuation of aluminum salt slags, finding that the metal oxide fraction can have direct applications as inert filler for construction, the paving of roads, and mortar components. Miqueleiz et al. [21] used alumina filler and coal fly ash waste as raw materials in the production of unfired bricks.

The objective of this research is to assess the possibility of using wastes from the aluminum secondary industry, aluminum filter dust, a residue not previously studied as a raw material, as a substitute for clay for the production of fired bricks. The influence of the waste content in the physical and mechanical properties was studied. The microstructure by SEM-EDS tests and an environmental study by TLCP (Toxicity Characteristics Leaching Procedure) leaching tests were also evaluated.

## 2. Materials and Methods

### 2.1. Materials

**Figure 1:**
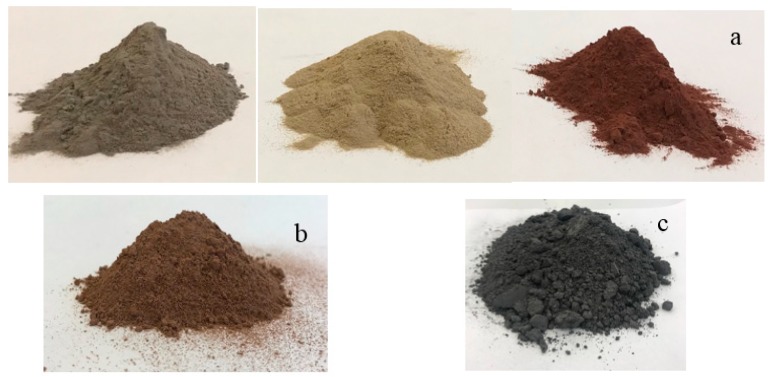
Photos of (**a**) the black, yellow, and red clay, (**b**) the clay mixture used as raw material, and (**c**) the aluminum filter dust waste (AFD).

Clay and aluminum filter dust (AFD) were used as raw materials. The clay was obtained from the clay quarries located in Bailén (Jaén, Spain). It is a mixture of three types of clays in different proportions: 40%, 30%, and 30% of black, red, and yellow clay, respectively (Figure 1a,b). The waste used is AFD (Figure 1c), generated in a blast furnace during the manufacture of secondary aluminum, and was a kind donation of Befesa Aluminio, S.L. (Valladolid, Spain).

### 2.2. Processing Method

The raw materials were dried in a stove at 105 °C for 24 h. Then, the clays underwent a process of grinding by means of a ball mill to crush them and reduce their particle size. Both the clay and the AFD were sieved until they reached a particle size <150 μm. Different proportions of AFD waste were added to the clay. The quantity used for both raw materials, as well as the nomenclature used for the different proportions are listed in Table 1. 

First, raw materials were weighted and mixed. To confer plastic properties to the mixture, 8 wt % of water was added to a kneader and mixed homogeneously. The obtained mixture was introduced in a rectangular matrix of size 60 mm × 30 mm × 10 mm. The ceramic pieces were obtained by compaction on a hydraulic press Mega Model KSC 15 (Melchor Gabilondo, S.A., Berriz, Spain) and shaped by exerting pressure of 10 MPa. The ceramic pieces were dried at 105 °C at 24 h using an oven in order to eliminate the moisture content and reduce the appearance of cracks. The specimens were fired using a Nabertherm furnace, with a heating rate of 2 °C/min and final temperature of 950 °C, maintaining this temperature for 1 h. Finally, the fired bricks were cooled down to room temperature. For comparative purposes, ten samples per series were produced. 

### 2.3. Techniques of Characterization of Raw Materials

The chemical composition of raw materials was determined by X-ray fluorescence (XRF). A Philips Magix Pro PW-2440 device (Amsterdam, The Netherlands) was used. The mineral phases of raw materials were determined by X-ray diffractometry (XRD) with an X’Pert Pro MPD automated diffractometer (PANanalytical, Westborough, MA, USA) equipped with a Ge (111) primary monochromator, using monochromatic Cu Kα radiation and an X’Celerator detector. The 2θ range was from 3° to 70°, step size at 0.03° (2 theta), scan speed at 0.05/240 (2 theta/s), and counting time at 240 s. The divergence slit was ½ (°theta) and antiscatter slit was ¼ (°2 theta). Thermogravimetric and differential thermal analysis (TGA-DTA) were performed with a Mettler Toledo 851e device (Columbus, OH, USA) in an oxygen atmosphere. 

### 2.4. Characterization of Fired Samples

The ceramic pieces obtained were subjected to a series of tests to verify if they comply with the regulations required to be used as construction materials. These tests included mass loss on ignition, linear shrinkage, bulk density, water absorption, water absorption by capillarity, and compressive strength. 

The linear shrinkage (%) was determined from the length of the samples before and after firing, using a caliper with a precision of ±0.01 mm, according to ASTM standard C326 [22]. The mass loss on ignition was determined as the mass loss between drying at 110 °C and firing at 950 °C. The bulk density (kg/m^3^) was determined according to the standard method UNE-EN 772-13: 2001 [23]. Water absorption (wt %) was determined according to the standard procedure UNE 772-21:2011 [24]. The water absorption by capillarity (kg/m^2^·min) was determined following the standard procedure UNE-EN 772-1:2001 [25]. The compressive strength was measured for six fired samples according to the standard procedure UNE-EN 772-1:2011 [26] in a Shimadzu laboratory testing equipment. 

Adsorption–desorption isotherms of N_2_ at 77 K, the specific surface area (BET), and micropore area were obtained in a Micromeritics equipment (TriStar II 3020 model, Micromeritics, Norcross, GA, USA). Then, the BJH method [27] was applied to determine the BJH cumulative volume of pores, BJH average pore diameter, and pore size distribution. 

The microstructure of brick samples was assessed by scanning electron microscopy (SEM) using a JEOL SM 840 (Akishima, Tokyo) equipped with energy dispersion spectroscopy (EDS). Samples were fractured and mounted on stubs using adhesive carbon pads and carbon coated before analysis. 

Finally, leaching tests of heavy metals in the samples were performed using the toxicity characteristic leaching procedure (TCLP), following the Environmental Protection Agency (EPA) method 1311 [28]. An inductively coupled plasma-atomic emission spectrometer (ICP-AES Agilent 7500, Santa Clara, CA, USA) was used to measure the concentrations in the filtrate.

## 3. Results and Discussion

### 3.1. Characterization of Raw Materials

The chemical composition of the clay mixture and the aluminum filter dust determined by XRF is shown in Table 2. High amounts of SiO_2_ (54.4 wt %), as predominant oxide, were detected in the raw clay, along with Al_2_O_3_ (12.36 wt %); this can be mainly attributed to aluminosilicates in the clay. The relevant proportion of CaO (8.76 wt %) could be derived from the high presence of carbonates, as indicated from the loss of ignition observed. The chemical composition of AFD showed considerable amounts of Al_2_O_3_ (21.56 wt %), and alkaline and alkaline earth oxides Na_2_O (21.69 wt %) y K_2_O (5.58 wt %) and, in a smaller quantity, Fe_2_O_3_ (0.64 wt %), CaO (1.31 wt %), and MgO (2.37 wt %). The large amount of flux oxides in the waste could decrease the firing temperature or the time of the ceramic bricks, saving energy in the process. Loss on ignition at 950 °C was 21.82%, probably attributable to a less effective combustion or thermal decomposition of inorganic species. 

**Figure 2:**
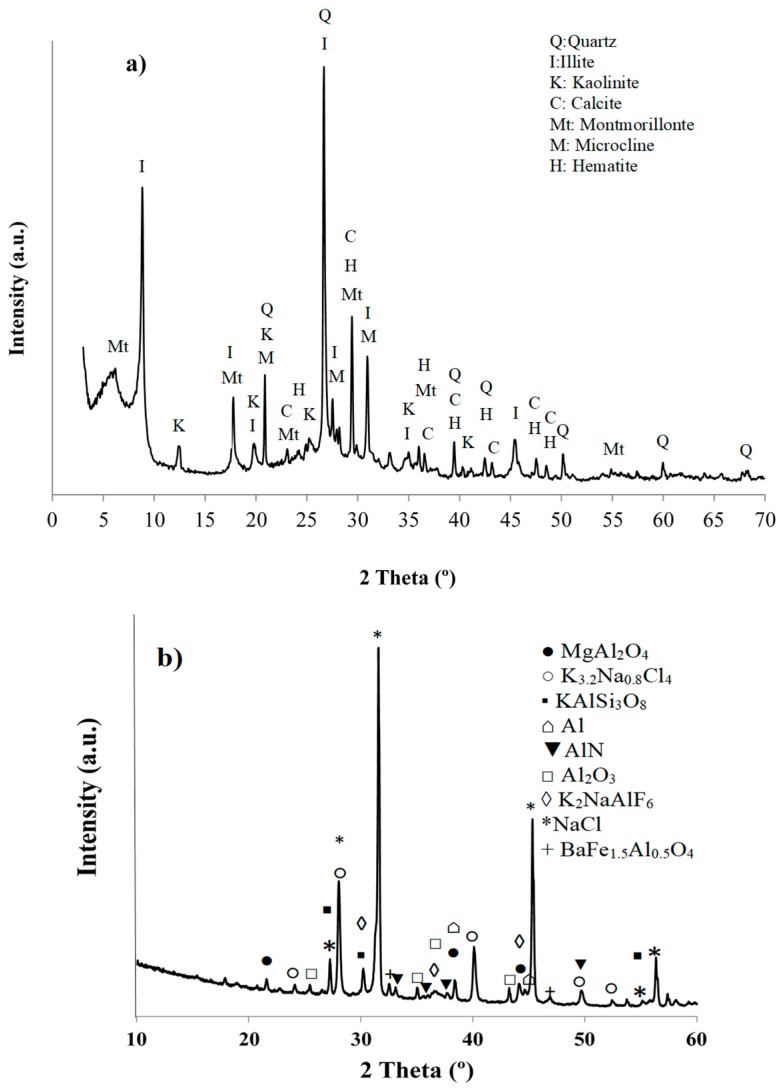
XRD pattern of (**a**) raw clay and (**b**) AFD waste.

The XRD pattern of the clay (Figure 2a) indicated that its mineralogical composition is composed of quartz (SiO_2_) and clayey minerals as kaolinite (Al_4_Si_4_O_10_(OH)_8_), aluminum potassium illite (KAl_2_(Si_3_Al)O_10_OH), and magnesium aluminum montmorillonite (MgOAl_2_O_35_SiO_2_xH_2_O). The raw clay also contained, in lower proportion, calcite (CaCO_3_), hematite (Fe_2_O_3_), and feldspars as microcline (KAlSiO_7_) and albite (NaAlSi_3_O_8_). 

The diffraction pattern of the AFD (Figure 2b) presents as main crystalline phases halite (NaCl), K_3.2_Na_0.8_Cl_4_, aluminum oxide (Al_2_O_3_), feldspars (silicoaluminate of alkaline and/or alkaline earth species) (KAlSi_3_O_8_), aluminum and magnesium spinel (MgAl_2_O_4_), elpasolite (K_2_NaAlF_6_), and BaFe_1.5_Al_0.5_O_4_.

**Figure 3:**
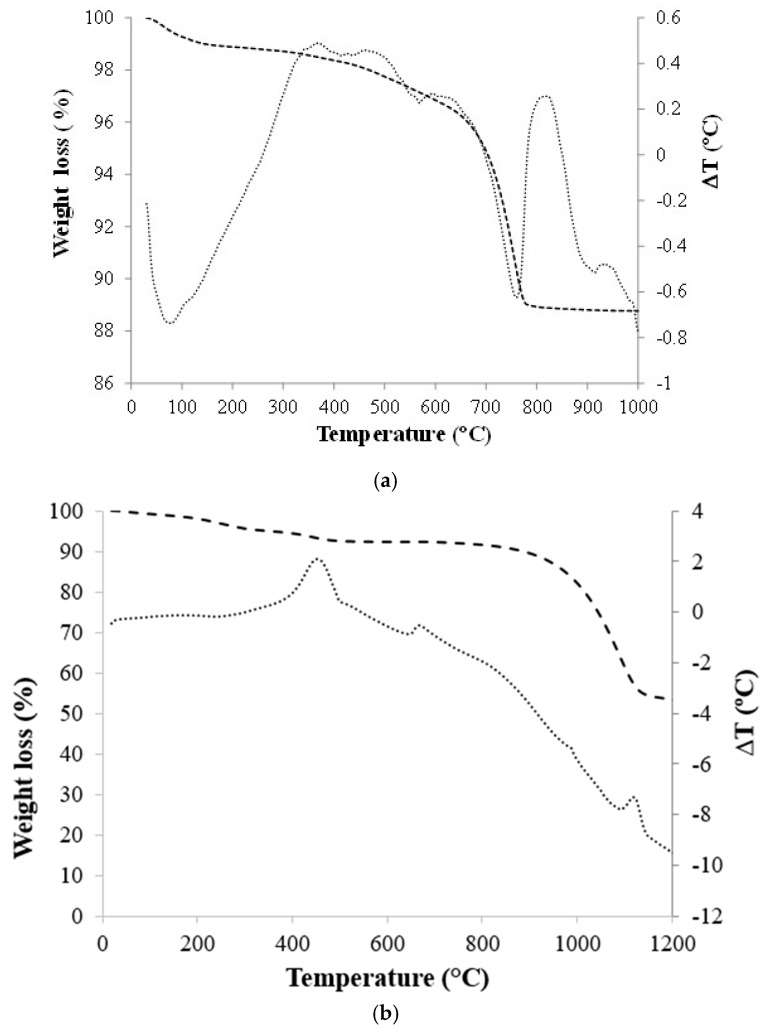
Thermogravimetric and differential thermal analysis (TGA-DTA) of (**a**) raw clay and (**b**) AFD waste.

The thermal behavior of the clay and the AFD residue is shown in Figure 3. Weight loss of 1% at temperatures between 30–150 °C was observed, with an endothermic peak centered at approximately 85 °C attributed to the loss of moisture. At temperatures between 150–600 °C, 2.2% weight loss was observed, associated with the combustion of organic matter and the dehydration of clay minerals, as indicated by the exothermic peaks centered at 375 and 475 °C and the endothermic peak centered at 570 °C, respectively. Between 600–800 °C, a weight loss of 8.2% was produced, assigned to the carbonates decomposition, as indicated by the endothermic peak centered at 760 °C. Finally, between 800–1000 °C, two exothermic peaks centered at 825 and 915 °C were observed, associated with the crystallization of the high temperature phases. The total weight loss of the clay was 11.2%.

The thermal behavior of the AFD waste is shown in Figure 3b. The total weight loss of the waste was 48.3%. The weight loss between 30–150 °C of 1.3% can be due to water evaporation (free and chemically bonded water). Between 150–375 °C, a weight loss of 4% was observed due to the combustion of organic matter, as indicated by the exothermic peak centered at 275 °C. The thermodifferential analysis curve presents two endothermic signals centered at 475 °C and 675 °C, the first one with a weight loss of 2.2% (375–500 °C) and the second one without weight loss, which could be due to crystallizations during the heat treatment. The greatest weight loss of 38% occurred between 750–1175 °C due to the carbonates decomposition and the crystallization of phases, such as the silicon and aluminum spinel prior to the mullite phase, as indicated by the exothermic peak at 775 °C and the endothermic peaks at 985 and 1130 °C.

### 3.2. Characterization of the Sustainable Fired Bricks

Following the firing process, no defects such as fissures, efflorescence, or bloating were observed. The color of the bricks with 100 wt % clay, control bricks, is a reddish orange color. As increasing percentages of AFD were added, the bricks become yellowish and tended to darken, showing a brown tone as the percentage of AFD increased. 

The linear shrinkage (Table 3) indicates the expansion/contraction behavior during the firing process. Linear shrinkage depends on the quantity of liquid phase produced in the firing process, as well as the decomposition of the gas phases. The linear contraction of the control brick was −2.88%, indicating an expansion. The addition of up to 20 wt % of waste hardly produced changes in the linear contraction after firing, producing a greater decrease when adding a 25 wt % of AFD reaching the linear contraction a value of −1.38%. These data indicate that both in the control bricks and in the bricks that incorporate the AFD residue, the release of gases due to the decomposition of organic matter, carbonates, and hematite predominated during the firing process.

The loss of ignition of the control brick at 950 °C was 12.89% (Table 3). The addition of increasing amounts of AFD waste produced an increase in the loss of ignition (LOI), from 13.82 with the addition of 5 wt % of residue to 20.22% with the addition of 25% residue, according to the highest LOI of the residue with respect to the clay (Table 2). The loss of ignition is due to the elimination of the water content of the clay mineral as a consequence of dehydroxylation reactions and the elimination of the organic matter content and the carbonates contained in the clay and the residue, as well as the decomposition of other inorganic components contained in the waste. 

**Figure 4:**
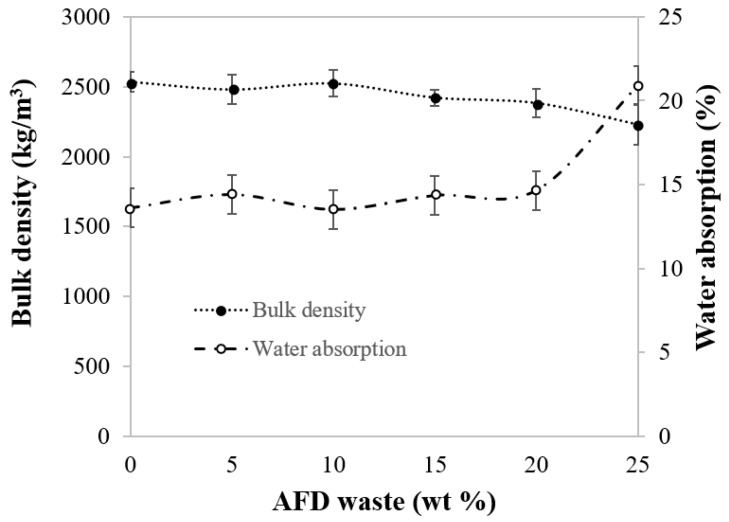
Bulk density and water absorption of fired bricks as a function of AFD waste content.

The addition of AFD reduced the bulk density of fired clay brick (Figure 4). The bulk density of the control bricks was 2534 kg/m^3^, decreasing by 2.17% and by 12% when 5 wt % and 25 wt % of AFD were added. This could be attributed to the lower relative density of the AFD residue compared to the clay. 

Water absorption by capillarity can significantly affect the durability and quality of the bricks. The bonding of the bricks with the mortar depends to a large extent on the water absorption by the capillarity capacity of the water in the brick and the water retention power of the mortar. Low values of water absorption of capillarity in the bricks contribute to a good durability and, consequently, greater resistance to the environment [29]. Water absorption by the capillarity of the control bricks was 2.127 kg/(m^2^ min) and increased slightly with the AFD waste addition (Table 3). Water absorption by capillarity increased from 2.437 kg/(m^2^ min) for 95C-5AFD bricks to 2.876 kg/(m^2^ min) for 75C-25AFD bricks, indicating an increase of 14.6% and 35.2%, respectively. The addition of AFD into the clay produced a higher interconnected surface porosity, due possibly to an increase in porosity, caused by the decomposition of organic matter and carbonate of the AFD waste in the firing stage. However, the values of water absorption by capillarity did not exceed 4.5 kg/(m^2^ min), which is the limit established by regulation RL-88 [30], according to which ceramic bricks must gather for their reception at construction. Bricks with higher values can cause dehydration of the mortar. For bricks with water absorption by capillary action greater than 1.5 kg/(m^2^ min), it is recommended to moisten them before they are laid. Thus, all bricks must be moistened to ensure proper mortar curing. 

Water absorption could be considered as an indirect measure of open porosity and a key factor for the durability of bricks. Water absorption of the control bricks was 13.6%. (Figure 4) The incorporation of up to 20 wt % of waste produced a slight increase in water absorption, reaching a value of 14.7%. Greater additions of AFD waste up to 25 wt % produced 75C-25AFD bricks with higher values of water absorption, increasing this property until it reached 20.9%. This increase could be a consequence of the chemical composition of the clay and the AFD residue. The clay and the residue have a content of flux materials (Na_2_O, K_2_O, MgO, and CaO) of 14.6% and 31.3%, respectively. The content of gaseous organic matter content was 2.29% and 8.9% and that of carbonates was 7.36% and 18.1%, respectively. The AFD waste contained a higher content of both flux material and gaseous compounds. The incorporation of the AFD waste into the clay body produced both the formation of molten material at a lower temperature that tends to compress the pores, and gaseous components, which generate gases and swell the ceramic bodies during the firing process. The results indicate that with the incorporation of up to 20 wt % of AFD, there is a balance between the fluxing effect and the pore-forming effect, increasing the pore-forming effect with higher additions, of 25 wt % of residue. According to ASTM C 67-03 [31], the maximum value allowed for severe weathering resistance ceramic bricks for construction is 22% and, hence, all the series produced comply with the standard. 

**Figure 5:**
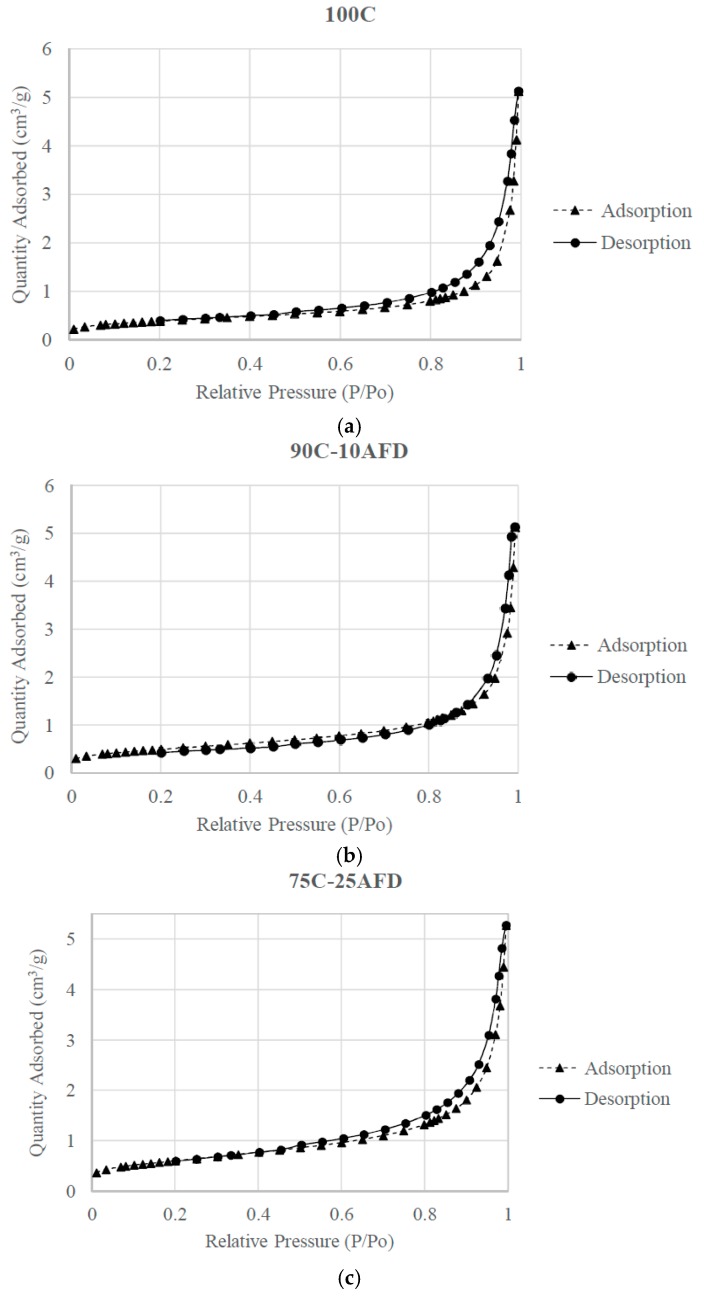
Adsorption-desorption isotherms of N_2_ at 77 K of fired clay bricks ((**a**) 100C; (**b**) 90C-10AFD and (**c**) 75C-25AFD).

The nitrogen adsorption isotherms (relative pressure, P/P_0_ versus volume adsorbed in cm^3^) are type IV adsorption isotherms, according to the IUPAC classification [32] (Figure 5); the hysteresis loop is typical of mesoporous materials. At low relative pressures, a low absorption was observed, with greater absorption at high pressures, which indicates that in these materials, the adsorption is carried out on the walls of the pores and is similar to the classic form observed in real porous solids or with structures where mesopores predominate. It is also worth noting that the addition of AFD residue produced a slight increase of the BET surface, from 1.41 m^2^/g for the control bricks to 2.19 m^2^/g for the 75C-25AFD, which indicates a greater number of pores in the body of clay with the incorporation of the residue. The area of micropores increased with the incorporation of the residue (Table 4).

**Figure 6:**
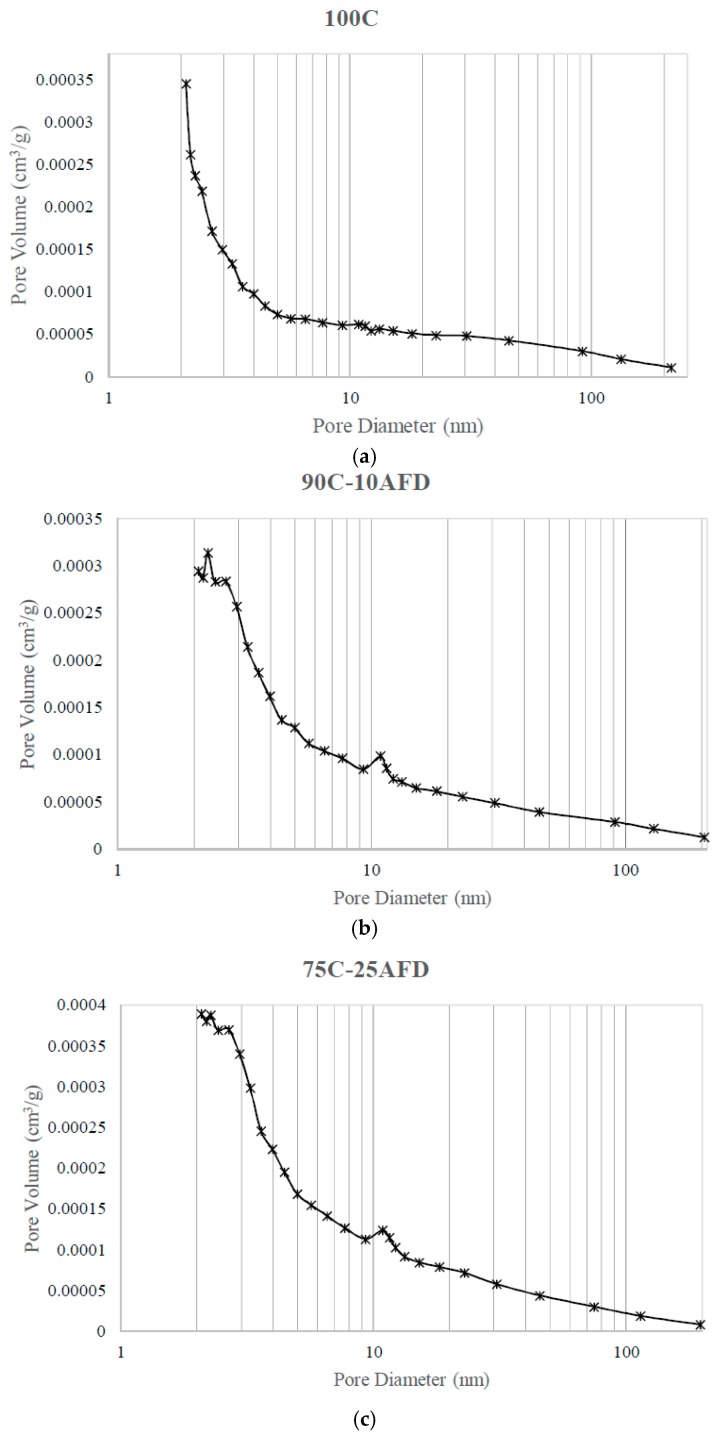
Pore size distribution determined from adsorption data using the BJH method for samples (**a**) 100C, (**b**) 90C-10AFD, and (**c**) 75C-25AFD.

The BJH model analyzes the pores between approximately 200 and 2 nm (mesopores). The graph of logarithmic pore diameter versus pore volume (Figure 6) showed that the control brick containing only clay has a heterogeneous pore distribution, presenting a maximum pore distribution of 24.65 nm. The mesoporous structure of the materials obtained was scarcely affected by the incorporation of AFD, presenting a slight decrease in pore size (Table 4).

**Figure 7:**
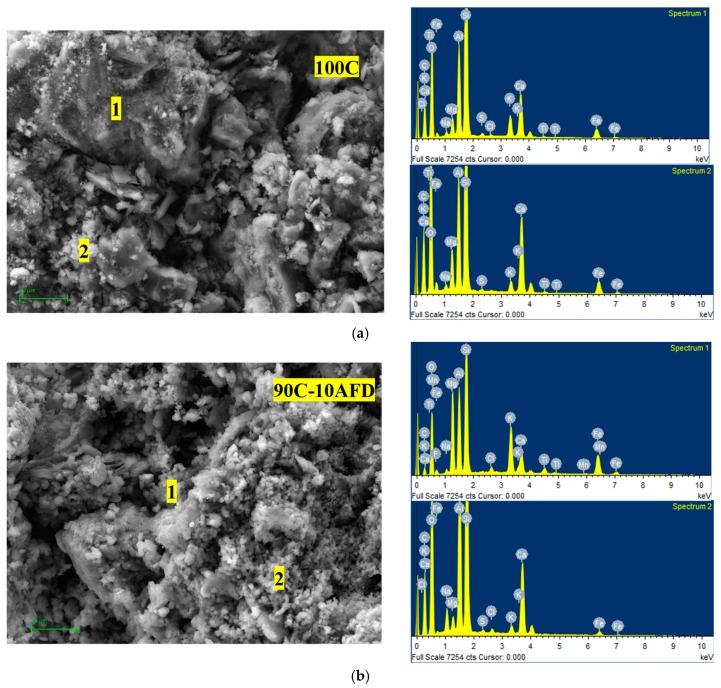

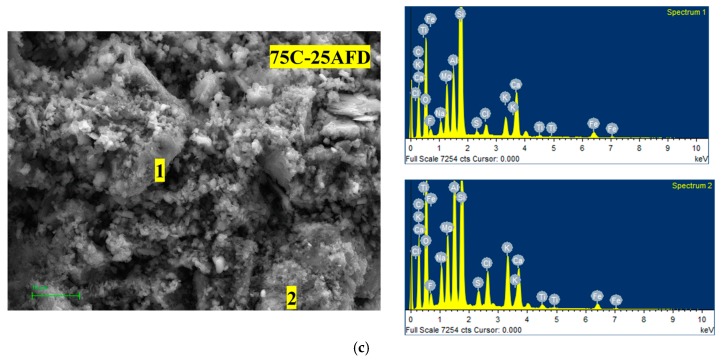
SEM micrograph with EDAX analysis at different points of (**a**) 100C, (**b**) 90C-10AFD, and (**c**) 75C-25AFD bricks.

The microstructure of fired bricks was studied by SEM coupled to chemical analysis by EDS (Figure 7). It is observed that both the control bricks and the bricks containing the AFD residue have a porous structure. The addition of different amounts of waste did not modify the composition of the bricks, as indicated by the EDAX analysis with regions rich in aluminosilicates; however, it modified the amount and size of the pores. The incorporation of waste produced an increase in the number of pores, decreasing the pore size to a greater extent with increasing quantities of waste. According to the water absorption and specific surface and pore size data, the addition of waste, due to its composition, produced both a fluxing effect and a pore-forming effect. During the firing process, the melting effect of the residue would produce a liquid phase during the firing process, which could close the internal pores and reduce the number and size of the pores, which would produce a slight densification of the brick. By contrast, the pore-forming effect of the residue would generate porosity in the body of the clay, producing an increase in porosity while the bulk density decreased. The incorporation of up to 20 wt % of waste produced a balance between both effects, predominating the pore forming effect with the addition of 25 wt % of AFD residue according to the data of bulk density, water absorption, and specific surface area.

**Figure 8:**
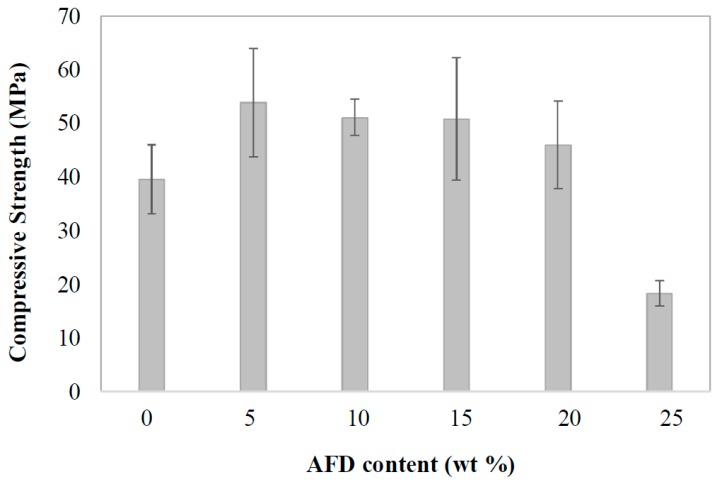
Compressive strength of fired bricks as a function of AFD waste content.

The compressive strength of ceramic materials is the most critical engineering property for building materials. The compressive strength of the fired samples is shown in Figure 8. The compressive strength values of the control brick was 39.6 MPa. The addition of up to 20 wt % in waste resulted in higher compressive strength values than that of the control brick, obtaining similar values of this property with the incorporation of up to 15 wt % of AFD waste (53–51 MPa). The incorporation of 25 wt % of waste produced bricks with a pronounced decrease in compressive strength of up to 18 MPa. The compressive strength depended on the porosity (number, type, and size of pores) in the body of the bricks. As can be deduced from the water absorption data, the open porosity of bricks containing up to 20 wt % of waste is similar to control bricks, with a slightly larger proportion of smaller pores and a slightly more compact structure. This distribution of porosity improved the compressive strength of the bricks. However, the addition of 25 wt % of residue produced a greater open porosity and a larger number of smaller pores that can act as stress concentrators, resulting in a lower compressive strength. However, the compressive strength values were always higher than the minimum value of 10 MPa required for RL-88 [30].

**Figure 9:**
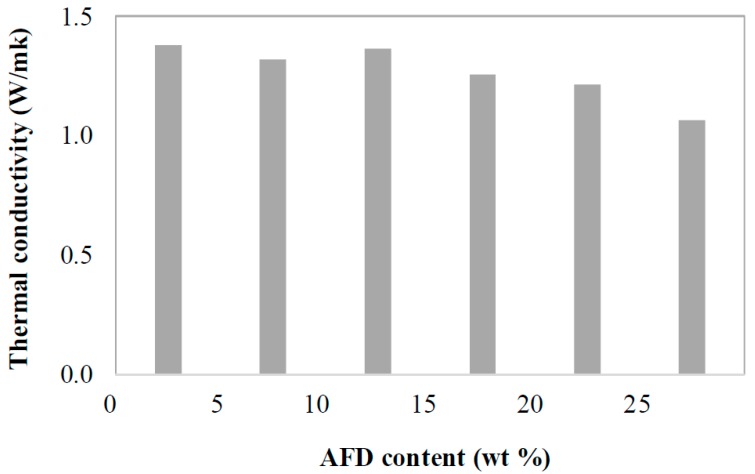
Estimated thermal conductivity of fired bricks as a function of AFD waste content.

Thermal conductivity is a key factor, since it determines the insulation capacity of construction bricks. Thermal conductivity is governed by bulk density as the most important factor in solids [33]. According to the data extracted from the Spanish standard NBE-CT-79 [34], the relationship between thermal conductivity and bulk density for bricks and blocks can be estimated by the expression:

k = 3 × 10^−7^ · ρ2 − 0.0004ρ + 0.4652

where k: Thermal conductivity (W/mK) and ρ: Bulk density. The thermal conductivity values decrease from 1.38 W/mK for the control brick to 1.22 and 1.07 with the incorporation of 20 and 25 wt % of AFD, which corresponds to a decrease of 12% and 23%, respectively, with respect to the thermal conductivity of the control brick without waste (Figure 9). This decrease in thermal conductivity with the addition of waste represents a significant energy saving that would favor the new requirements for thermal insulation in new buildings.

Table 5 shows the results of the heavy metal leaching tests of the fired bricks that incorporate different contents of AFD waste. In general, the concentration of heavy metals increased slightly with the incorporation of the waste. The addition of up to 25 wt % of AFD produced an increase of 0.3 ppb to 24.7 ppb of Cu and 0.3 to 1.3 of Zn. However, these values are much lower than those established by the US Environmental Protection Agency (USEPA) limits, 5000 ppb for Cu and 300 ppb for Zn (Table 5). It has been observed that the incorporation of AFD waste produced an increase in surface area; this increase in the contact area between the bricks and the leaching solution could produce a slight increase in the leaching of heavy metals. However, leaching test data indicate that heavy metals have been effectively immobilized during the firing process. This allows bricks to be classified as acceptable at landfills for inert and nonhazardous waste.

## 4. Conclusions

In this work, filter dust from the aluminum industry was used as raw material for the manufacture of clay bricks. The waste composition is rich in aluminum and also has a high percentage of fluxes and gaseous compounds. The incorporation of increasing amounts of AFD produced bricks with a greater specific surface, that is, with a larger number of pores, but smaller in size. Adding up to 20 wt % produced bricks with a bulk density and water absorption similar to that of the control brick, with a higher compressive strength and lower thermal conductivity, which may be due to the balance between the melting effect and the pore-forming effect of the residue. Higher additions of 25 wt % of the residue produced bricks with lower bulk density and compressive strength and higher values of water absorption and specific surface area, predominating the pore-forming effect. The leaching test data indicate that an effective immobilization of heavy metals is achieved for all the bricks studied with the incorporation of up to 25 wt % of waste.

Therefore, the results indicate that up to 20 wt % of waste can be incorporated, obtaining bricks with physical properties similar to conventional bricks and improved mechanical and thermal properties.

The bricks of this study, due to the physical and mechanical properties, could be manufactured as solid bricks or with holes. The bricks can be used as facing bricks, uncoated, providing an interior or exterior finish, as well as coated bricks, being structural elements that can bear loads. In addition to the technical characteristics, the AFD bricks present economic and environmental benefits that would result from the recovery of this waste by eliminating the amount of waste deposited in landfills.

## Figures and Tables

**Table 1:** Mixture proportions of raw materials for brick manufacturing.

Brick Series	Clay (wt %)	FDA (wt %)
100C	100	-
95C-5FDA	95	5
90C-10FDA	90	10
85C-15FDA	85	15
80C-20FDA	80	20
75C-25FDA	75	25

**Table 2:** Chemical composition of clay and aluminum filter dust.

Oxide Content (%)	Clay	AFD
SiO_2_	54.4	0.38
Al_2_O_3_	12.36	21.56
Fe_2_O_3_	4.58	0.64
CaO	8.76	1.31
MgO	2.46	2.37
MnO	0.03	0.05
Na_2_O	-	21.69
K_2_O	3.37	5.88
TiO_2_	0.60	0.37
P_2_O_5_	0.11	0.03
SO_3_	0.68	-
ZnO	0.026	-
SrO	0.027	-
ZrO_2_	0.033	-
Cl	-	24.42
LOI	12.51	21.82

**Table 3:** Technological properties of fired bricks made from clay and AFD mixtures.

Sample	Linear Shrinkage (%)	Mass Loss on Ignition (%)	Suction Water (kg/m^2^·min)
100C	−2.878 ± 0.654	12.890 ± 0.049	2.127 ± 0.217
95C-5FDA	−3.092 ± 0.673	13.818 ± 0.548	2.437 ± 0.068
90C-10FDA	−2.720 ± 0.134	13.894 ± 0.326	2.592 ± 0.114
85C-15FDA	−2.800 ± 0.175	15.388 ± 1.098	2.623 ± 0.159
80C-20FDA	−2.827 ± 0.140	16.105 ± 0.985	2.782 ± 0.262
75C-25FDA	−1.379 ± 0.397	20.216 ± 0.918	2.876 ± 0.152

**Table 4:** BET surface, micropore area, BJH cumulative volume of pores, and BJH average pore diameter for clay-aluminum filter dust bricks.

Sample	BET Surface Area (m^2^/g)	t-Plot Micropore Area (cm^3^/g)	BJH Cumulative Volume of Pores (between 1.7 and 300 nm) (cm^3^/g)	BJH Average Pore Diameter (nm)
100C	1.4134	0.1302	0.008012	24.6500
95C-5FDA	1.5759	0.2408	0.007365	21.8648
90C-10FDA	1.7654	0.2002	0.008023	19.0877
85C-15FDA	1.8653	0.1975	0.006574	14.6692
80C-20FDA	1.8797	0.2799	0.007348	17.5119
75C-25FDA	2.1905	0.2249	0.008291	15.6814

**Table 5:** Toxicity characteristic leaching procedure (TCLP) leaching test results (ppb) in the fired samples with different AFD contents and the maximum concentration (Environmental Protection Agency (EPA) regulated TCLP limits) of contaminants for toxicity characteristics.

Component (ppb)	100C	95C-5AFD	90C-10AFD	85C-15AFD	80C-20AFD	75C-25AFD	USEPA Regulated TCLP Limits (ppb)
V	0.990	3.062	1.146	1.194	1.870	0.829	N/A
Cr	0.021	0.251	0.371	0.188	0.393	0.493	5000
Co	0.714	0.242	0.153	0.673	0.469	0.030	N/A
Ni	0.151	0.463	0.110	0.128	0.083	0.047	250
Cu	0.310	2.338	9.291	12.210	14.185	24.698	5000
Zn	0.321	1.088	0.724	0.783	0.304	1.323	300
As	0.024	0.042	0.004	0.007	0.017	0.003	5000
Cd	0.026	0.016	0.155	0.177	0.247	0.351	1000
Ba	0.295	0.489	0.498	0.562	0.565	0.591	100
Hg	0.000	0.000	0.000	0.000	0.000	0.000	200
Pb	0.000	0.020	0.076	0.000	0.000	0.013	5000
